# Prevention of childhood overweight and obesity in Mongolia, the Philippines and Vietnam: identifying priority actions

**DOI:** 10.1093/heapro/daad187

**Published:** 2023-12-29

**Authors:** Bolormaa Norov, Cherry Cristobal-Maramag, Hoang Van Minh, Khương Quỳnh Long, Oliver Huse, Alice Nkoroi, Munkhjargal Luvsanjamba, Do Hong Phuong, Roland Kupka, Tim Lobstein, Jo Jewell, Mary Christine Castro, Nikka Oliver, Fiona Watson

**Affiliations:** Nutrition Department, National Center for Public Health, Peace Ave 46, Ulaanbaatar 13381, Mongolia; Health and Nutrition Unit, Nutrition Center of the Philippines, Launchpad Coworking 214-215 Commercenter, East Asia Drive cor. Commerce Avenue, Filinvest Corporate City, Alabang Muntinlupa City, The Philippines; Hanoi University of Public Health, 1A Đ. Đức Thắng, Đông Ngạc, Bắc Từ Liêm, Hà Nội, Vietnam; Hanoi University of Public Health, 1A Đ. Đức Thắng, Đông Ngạc, Bắc Từ Liêm, Hà Nội, Vietnam; Deakin University, Geelong Australia, Global Obesity Centre for Preventive Health and Nutrition, Institute for Health Transformation, 1 Gheringhap St 3220; East Asia and Pacific Regional Office, UNICEF, 19 Pra Athit Rd, Chana Songkhram, Pra Nakhon, Bangkok 10200, Thailand; Philippines Country Office, UNICEF, 14th Floor, North Tower, Rockwell Business Center Sheridan, Sheridan Street corner United Street, Highway Hills, Mandaluyong City 1550, Philippines; Mongolia Country Office, UNICEF, UN House, United Nations street-14, Ulaanbaatar 14201, Mongolia; Vietnam Country Office, UNICEF, The Green One UN House, 304 Kim Ma, Ba Dinh District, Hanoi, Vietnam; East Asia and Pacific Regional Office, UNICEF, 19 Pra Athit Rd, Chana Songkhram, Pra Nakhon, Bangkok 10200, Thailand; Policy Section, World Obesity Federation, 5th Floor, 38 Chancery Lane, London, WC2A 1EN, UK; The Boden Initiative, University of Sydney, John Hopkins Dr, Camperdown, Sydney, 2050, NSW, Australia; Nutrition Section, UNICEF, 3 United Nations Plaza, New York, NY 10017, USA; Health and Nutrition Unit, Nutrition Center of the Philippines, Launchpad Coworking 214-215 Commercenter, East Asia Drive cor. Commerce Avenue, Filinvest Corporate City, Alabang Muntinlupa City, The Philippines; Health and Nutrition Unit, Nutrition Center of the Philippines, Launchpad Coworking 214-215 Commercenter, East Asia Drive cor. Commerce Avenue, Filinvest Corporate City, Alabang Muntinlupa City, The Philippines; East Asia and Pacific Regional Office, UNICEF, 19 Pra Athit Rd, Chana Songkhram, Pra Nakhon, Bangkok 10200, Thailand

**Keywords:** childhood overweight, nutrition policy, food environments, public health, Southeast Asia

## Abstract

Low- and middle-income countries are increasingly faced with a triple burden of malnutrition: endemic underweight, micronutrient deficiencies and rising prevalence of overweight. This study aimed to address existing knowledge gaps and to identify priority policy options in Mongolia, the Philippines and Vietnam. A landscape analysis approach was adopted using methods set out in a UNICEF global toolkit. Quantitative and qualitative data were compiled from a range of global and national sources on childhood overweight and obesity, risk factors and policy responses. Key informant interviews and validation workshops were undertaken with key food and nutrition stakeholders from government and non-government organizations to identify priority policy options for the prevention of overweight and obesity among children. Overweight and obesity among children are increasing in all three countries. Associated risk factors are related to maternal nutrition, birthweight, breastfeeding, as well as diets and physical activity shaped by increasingly obesogenic environments. Key informants identified undefined policy approaches, poor community understanding and food and beverage industry influence as barriers to addressing overweight and obesity. Key policy priorities include restricting the marketing of unhealthy food and beverages, unhealthy food and beverage taxation, introduction of front-of-pack nutrition labels and improving school nutrition environments. Mongolia, the Philippines and Vietnam are all facing an increasing burden of childhood overweight and obesity. Despite differing national contexts, similar environmental factors are driving this rise. A suite of evidence-based policies can effectively be introduced to address obesogenic environments.

**Contribution to Health Promotion Statement**

- This landscape analysis into childhood overweight and obesity in Mongolia, the Philippines and Vietnam aimed to address the existing knowledge gaps and to identify the priority policy options in these countries.
- We found that overweight and obesity among children are increasing in all the three countries, and increasingly obesogenic environments are driving the risk factors for overweight and obesity.
- Key priorities for action to prevent the overweight and obesity focussed on improving the food environment.
- Our study provides governments and other key stakeholders with robust evidence for strengthening policy actions to improve food environments and address growing rates of childhood overweight and obesity.

## INTRODUCTION

For many low- and middle-income countries (LMICs), undernutrition, in the form of stunting, wasting and micronutrient deficiencies, is still a major health concern (Victora *et al.*, 2021). This has resulted in significant morbidity and mortality and makes a compelling case for the implementation of policies to address undernutrition (Black *et al.*, 2008). However, LMICs are also facing the rising rates of overweight and obesity, leading to a triple burden of malnutrition (Yang *et al.*, 2019). The triple burden of malnutrition has substantive health and social consequences, placing added pressure on health and societal systems (Nugent *et al.*, 2020; Wells *et al.*, 2020).

In Asia, 17% of children aged 5−11 years and 23.2% of adolescents aged 12−19 years were estimated to be living with overweight or obesity in 2018 (Mazidi *et al.*, 2018). These children are at a higher risk of developing a range of non-communicable diseases (NCDs) and may experience psychological and psychosocial impacts (Daniels, 2009). They are also more likely to be affected by overweight and obesity later in life, heightening the risk of NCDs and premature mortality (Daniels, 2009). Despite the rising rates of childhood overweight and obesity, many of these countries are still facing high rates of stunting, wasting and micronutrient deficiencies, indicating a triple burden of malnutrition (Haddad *et al.*, 2014). Mongolia, Philippines and Vietnam are the three LMICs (Gross National Income ≤ USD 1,045 per capita) in the East Asia region (World Bank, 2019), where a growing prevalence of childhood overweight and obesity has been reported (Lobstein *et al.*, 2022).

The environments in which children live and grow up are driving overweight and obesity. Obesogenic environments are those where unhealthy foods and beverages are more available, affordable and promoted, and where opportunities for physical activity are reduced (Osei-Assibey *et al.*, 2012). Early life environments, even during the gestational period, affect behavioural and biological responses, predisposing an individual to overweight and obesity over the course of their lives (Ziauddeen *et al.*, 2018). The actions of the commercial food and beverage sector have the potential to influence food environments to favour unhealthy diets through unhealthy food and beverage marketing, supply chain and food systems activities, and political lobbying to influence food and nutrition policies—a concept known as the commercial determinants of health (Stuckler and Nestle, 2012; Moodie *et al.*, 2021).

Strong evidence supports the notion that governments and government policy can support populations to consume healthy diets and lead healthy lives (Kickbusch, 2015). Globally, there is increasing interest in policies and strategies that directly target obesogenic environments and counter the actions of unhealthy food and beverage corporations, which are known to be effective (World Health Organization, 2016, 2017). Such strategies include taxation of unhealthy foods and beverages, restrictions on the marketing of unhealthy foods and beverages to children, restrictions on the marketing of breastmilk substitutes (BMS), front-of-pack nutrition labelling (FOPNL) and regulations on the foods and beverages that are available in schools (World Health Organization, 2016, 2017). Despite this, progress in tackling childhood overweight and obesity has been slow and inconsistent.

In Asia, the focus of nutrition research, policies and programmes has largely been on the prevention of undernutrition in the form of stunting, wasting and micronutrient deficiencies among under-fives (Haddad *et al.*, 2014; Khandelwal and Kurpad, 2019). There has been less focus on the overweight and obesity as prevalence rates remain relatively low in this age group. Among older children, the prevalence rates are rising sharply, but the absence of global targets and limited availability of survey data means that less attention has been directed at addressing overweight and obesity in this cohort (Haddad *et al.*, 2014; Khandelwal and Kurpad, 2019).

Health promotion represents a complex challenge to decision makers, but these policymakers should be held to account to their role in policy formulation and implementation (Thomas and Daube, 2023). As governments continue to grapple with undernutrition, there is a growing need to ensure that policies and programmes are also addressing the rising burden of childhood overweight. Governments need to have clarity on the extent of this problem, the causes, and effective policies and programmes. This landscape analysis aimed to synthesis the existing evidence on the prevalence and trends in childhood overweight and obesity and related risk factors and describe the policy responses to this, in Mongolia, Philippines and Vietnam.

## METHODS

This country-level landscape analysis was structured according to a global toolkit [United Nations Children’s Fund (UNICEF), 2022]. This novel approach was developed by UNICEF in response to calls from country offices for guidance as to how to better understand the childhood overweight and obesity situation in a given country context. The toolkit contains five parts to be followed:

1. Assess the prevalence and trends in overweight and obesity among children.
2. Review key risk factors for overweight and obesity among children.
3. Review policies that influence overweight and obesity among children.
4. Assess policy options for prevention of overweight and obesity among children.
5. Agree on policy priorities for prevention of overweight and obesity among children.

The landscape analysis methods included compilation of quantitative and qualitative data from a range of global and national sources, key informant interviews and a validation workshop. National staff members from public health institutions in Mongolia, Philippines and Vietnam were responsible for data collation and analysis. Mongolia, Philippines and Vietnam were selected for the participation in this analysis based on expressions of interest from UNICEF country office staff.

1. *Prevalence and trends in overweight and obesity among children*

Data for children aged < 5 years was taken from the UNICEF/World Health Organization (WHO)/World Bank Joint Malnutrition Estimates (United Nations Children’s Fund, World Health Organization *et al.*, 2012). Overweight is defined as weight-for-height more than 2 standard deviations (SD) above the median of the WHO Child Growth Standards reference population (WHO Multicentre Growth Reference Study Group, 2006). Data for children aged 5−19 years was taken from Non-communicable Risk Factor Collaboration (NCD-RisC) (NCD Risk Factor Collaboration, 2017). Overweight was defined as a body mass index-for-age *Z*-score (BMIZ) of 1 to <2 SD and obesity as BMIZ of ≥2 SD above the WHO Growth Reference median (de Onis *et al.*, 2007). Both the UNICEF/WHO/World Bank Joint Malnutrition Estimates (United Nations Children’s Fund *et al.*, 2012) and the NCD-RisC (NCD Risk Factor Collaboration, 2017) are described in greater detail in Supplementary File 1.

Analysis of quantitative data was descriptive. Results are presented for children aged under 5 years (with boys and girls combined) and for boys and girls aged 5−19 years separately between 2000 and 2016. Published thresholds are used to classify the reported prevalence of childhood overweight and obesity as ‘high’, ‘moderate’ and ‘low’ (Lobstein and Jewell, 2021).

2. *. Key risk factors for overweight and obesity among children*

The included risk factors for overweight and obesity among children are presented in Tables 1 and 2 and Supplementary File 2. Selection was based on (i) the strength of the evidence to demonstrate an association with overweight and obesity in children and (ii) availability of consistent cross-country data sources. Information on risk factors was collected from a range of global and national surveys and academic publications. These included the Global School-Based Student Health Surveys (GSHS) (World Health Organization., 2022b), NCD-RisC data (NCD Risk Factor Collaboration, 2017), the UNICEF Infant and Young Child Feeding (IYCF) Database (United Nations Children’s Fund and World Health Organization, 2021), UNICEF/WHO Low Birthweight Estimates (United Nations Children’s Fund and World Health Organization, 2019), UNICEF/WHO/World Bank Joint Malnutrition Estimates (United Nations Children’s Fund *et al.*, 2012), the WHO Global Health Observatory (World Health Organization, 2022a) and the WHO/UNICEF Joint Monitoring Programme for Water Supply, Sanitation and Hygiene (WASH) (World Health Organization and United Nations Children’s Fund, 2021). All relevant databases listed herein are described in greater detail in Supplementary File 1.

**Table 1:** Prevalence of risk factors for the development of overweight and obesity among children aged 0–5 years

	Mongolia		Philippines		Vietnam	
Indicator	Value	Threshold	Value	Threshold	Value	Threshold
Prenatal						
Prevalence of obesity among women of reproductive age (15−49 years old)	16.5%^1^	Moderate	35.2%^6^	Poor	24.0%^7^	Moderate
Prevalence of tobacco smoking among women of reproductive age (15−49 years old)	6.4%^1^	Moderate	2.3%^6^	Good	1.1%^8^	Good
Postnatal						
Births with a reported birth weight below 2.5 kg	4.6%^2^	Good	14%^6^	Poor	8.2%^2^	Moderate
Births with a reported birth weight above 4.0 kg	12.6%^3^	Poor	11.2%^6^	Poor	2.3%^9^	Good
Stunting						
Stunting in children under 5 years old	9.4%^4^	Good	28.8%^6^	Poor	19.6%^10^	Moderate
Infant and young child feeding						
Infants who were not initiated to breastfeed within 1 hour after delivery	29.8%^5^	Moderate	26%^6^	Good	35.0%^10^	Moderate
Infants 0−5 months who were not fed exclusively with breast milk	49.8%^5^	Moderate	42.1%^6^	Moderate	54.6%^10^	Moderate

^1^WHO Global Health Observatory.

^2^UNICEF/WHO Low Birthweight Estimates.

^3^Ministry of Health, National Center for Public Health, UNICEF. 2017. The nutrition status of the population of Mongolia.

^4^UNICEF/WHO/World Bank Joint Malnutrition Estimates.

^5^UNICEF Infant and Young Child Feeding Data.

^6^DOST-FNRI. (2020). Expanded National Nutrition Survey: 2019.

^7^NCD Risk Factor Collaboration. National adult body-mass index. https://ncdrisc.org/data-downloads-adiposity.html.

^8^MoH of Viet Nam, Hanoi Medical University, General Statistics Office, CDC, WHO. Viet Nam Global Adult Tobacco Survey 2015. Hanoi, Vietnam.

^9^National Institution of Nutrition. National Nutrition Strategy for 2015. Hanoi, Vietnam, 2015.

^10^National Institution of Nutrition. National Nutrition Strategy for 2020. Hanoi, Vietnam, 2020.

**Table 2:** Prevalence of risk factors for the development of overweight and obesity among children aged 5−19 years

	Mongolia		Philippines		Vietnam	
Indicator	Value	Threshold	Value	Threshold	Value	Threshold
Dietary risk factors						
Proportion of all children consuming sugary drinks at least once per day	34.0%^1^	Moderate	37.6%^4^	Moderate	34.9%^6^	Moderate
Proportion of all children consuming fast food at least once per week	54.9%^1^	Poor	51.3%^4^	Poor	17.1%^6^	Moderate
Fruit and vegetable consumption among children	81.1% consumed <5 serves of fruits or vegetables per day^1^	Poor	80.8% consumed < 3 serves of fruit per day^4^ 73.6% consumed < 3 serves of vegetables per day^4^	Poor Poor	83.1% consumed < 3 serves of fruit per day^7^ 80.7% consumed < 3 serves of vegetables per day^7^	Poor Poor
Proportion of households without access to safe drinking water	14.5%^2^	Moderate	5.9%^2^	Good	3.1%^2^	Good
Proportion of schools without access to safe drinking water	25.6%^2^	Moderate	53.1%^2^	Poor	—	—
Physical activity risk factors						
Children and youth aged 11–17 years old not meeting WHO recommendations on Physical Activity for Health	78.8%^3^	Poor	93.4%^3^	Poor	86.3%^3^	Poor
Students aged 13–17 years old who spent three or more hours per day engaged in sedentary behaviours	71.8%^1^	Moderate	31.9%^4^	Moderate	59.3%^6^	Moderate
Proportion of children who did not use active transport to travel to school	44.3%^1^	Moderate	—	—	41%^6^	Moderate
Proportion of children who routinely had <8 h of sleep per night	—	—	12.7% (private school children)^5^ 13.8% (public school children)^5^	Good Good	43.0%^8^	Poor

^1^Global School-Based Student Health Survey—Mongolia, 2013.

^2^ World Health Organization and United Nations Children’s Fund, (2021). State of the World’s Children 2019.

^3^World Health Organization. Global Health Observatory data repository—prevalence of insufficient physical activity among school going adolescents. 2016.

^4^Global School-Based Student Health Survey—Philippines, 2015.

^5^Florentino, R.; Villavieja, G.; & Lana, R. (2006). Dietary and physical activity patterns of 8- to 10-year-old urban schoolchildren in Manila, Philippines. Food and Nutrition Bulletin (23, 3), pp. 267−273.

^6^Global School-Based Student Health Survey—Vietnam, 2019.

^7^Global School-Based Student Health Survey—Vietnam, 2013.

^8^Carrillo-Larco RM, Bernabé-Ortiz A, Miranda JJ. Short sleep duration and childhood obesity: cross-sectional analysis in Peru and patterns in four developing countries. PLoS One. 2014;9(11):e112433.

Analysis of quantitative data on risk factors for the development of childhood overweight and obesity are descriptive. Thresholds, developed for use with the UNICEF global toolkit, were used to classify the risk factors for overweight and obesity as ‘poor’, ‘moderate’ and ‘good’ (United Nations Children’s Fund (UNICEF), 2022).

3. *Policies that influence overweight and obesity among children*

A range of government and global databases were searched to identify the presence or absence of key policies. The full set of policies assessed are in Supplementary File 3. Data on six key policy areas are presented in Table 3. These have been consistently recommended by international authoritative health bodies as critical to address overweight, obesity and unhealthy diets among the children (World Health Organization, 2016, 2017):

**Table 3:** Implementation of key policies to address childhood overweight and obesity in Mongolia, the Philippines and Vietnam

Policy	Mongolia	Philippines	Vietnam
National policy on prevention of overweight and obesity in children	No specific policy on overweight and obesity though targets for overweight reduction for children are included in the Mongolian National Programme on Nutrition 2016–2025	No specific policy on overweight and obesity though targets for overweight reduction for children are included in the Philippine Plan of Action for Nutrition (PPAN) 2017–2022	Targets for childhood overweight and obesity are included in the current National Nutrition Strategy
Tax on sugar-sweetened beverages	None	Republic Act 10963 ‘Tax Reform for Acceleration and Inclusion (TRAIN) Law’ (2018) imposed a tax of PhP6.00 or PhP12.00 on sweetened beverages depending on sugar content	None, though a 10% tax on sugar-sweetened beverages is propose
Controls on marketing of food and non-alcoholic beverages to children	None	Voluntary ‘Philippine Responsible Advertising to Children Pledge’ signed by 12 big food and beverage manufacturers ‘Policy and Guidelines on Healthy Food and Beverage Choices in Schools’ (2017) prohibits the promotion of unhealthy foods and beverages in schools	None
Controls on marketing of breast milk substitutes	Control on the promotion and marketing of BMS regulated by Mongolian law on foods for infants and toddlers endorsed in July 2017	Executive Order No. 51 or ‘Philippine Milk Code’ (1986) recognizes the international code on the marketing of breastmilk substitutes	Government Decree 100 on marketing and use of nutrition products for young children (2014) regulates the ban on advertising of BMS for young children under 24 months and complementary foods for children under 6 months
Front-of-pack nutrition labelling	Health Ministerial order # 221, 2017 encourages food manufacturers to include a colour coded traffic light FOPNL dictating calorie, total fat, saturated fat, sugar and salt content of a product	Only voluntary ‘healthier choice’ logo, which does not identify less-healthy options	None
School nutrition environments	The Mongolian parliament endorsed the ‘Law on Secondary school catering service’ (2019). In 2021 the health minister enforced order #A370, ‘List of food products to prohibit sale in school environment’. The list includes highly processed foods	‘Policy and Guidelines on Healthy Food and Beverage Choices in Schools’ (2017) prohibits the sale of unhealthy foods and beverages in schools, though adoption of this element of the policy is voluntary	Government school meals project funded by the Ajinomoto Viet Nam company is currently being rolled out nationwide. The school milk programme was approved through Decision No. 1340 (2016). Milk selection school/parent-dependant and includes flavoured or sweetened milk. There is no goal related to control childhood overweight and obesity

1. National policy on prevention of overweight and obesity in children
2. Sugar-sweetened beverage taxation
3. Unhealthy food and non-alcoholic beverage marketing restrictions
4. BMS marketing restrictions
5. FOPNL
6. School nutrition environments

Data analysis was descriptive, with both the presence and the absence of key policies identified.

4. *Policy options for prevention of overweight and obesity among children*

Key informant interviews were conducted with two purposes. First, to ascertain whether the findings from the first three stages of the landscape analysis accorded with interviewees’ perceptions. Second, to identify the key strengths and gaps in policies and programmes to address overweight and obesity in children as well as the key barriers to their implementation.

A convenience sampling approach was adopted to recruit key informants, in local and national government departments, non-government organisations, professional societies and academic institutions. The interviews took ~45 min to complete and were conducted in the respective national language. Interview questions were informed by the UNICEF landscape analysis global toolkit (United Nations Children’s Fund (UNICEF), 2022).

All interviews were conducted with the intention of informing policy and policymakers and were conducted by in-country consultants. Following the conclusion of interviews, these in-country consultants summarised the interviews and identified key quotes. The fourth author, who has a background in the health promotion and food and nutrition policy, constructed key overall themes from the data summaries from the three countries. Trustworthiness of data was ensured through validation workshops where landscape analysis results were discussed.

5. *Policy priorities for prevention of overweight and obesity among children*

Validation workshops, hosted by UNICEF and national government partners were conducted in Mongolia and the Philippines prior to May 2021, but not in Vietnam, due to the COVID-19 pandemic. The aim of the workshops was fourfold. First, to validate the results from the first four stages of the landscape analysis approach with key in-country stakeholders. Second, to communicate the extent of childhood overweight and obesity. Third, to ensure that participants were aware of existing programmes, policies and gaps. Fourth, to identify possible policies, programmes and interventions to address the emerging scale of childhood overweight and obesity.

Participants were key stakeholders in national government agencies, professional organizations and partner institutions. The methods and findings of the landscape analyses were presented first, followed by breakout groups in which participants discussed the major evidence and policy gaps, and identified feasible priority actions.

## RESULTS

### Prevalence and trends in overweight and obesity among children

**Fig. 1:**
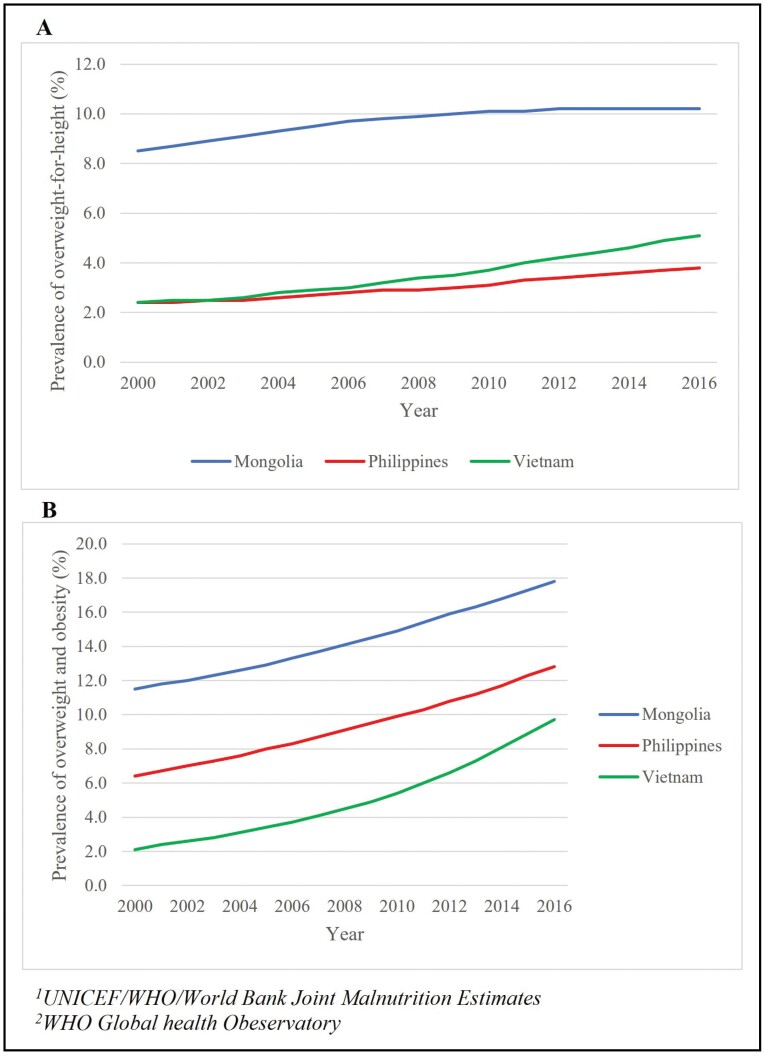
Prevalence of childhood overweight and obesity in Mongolia, the Philippines and Vietnam.

The prevalence of overweight among children aged <5 years increased between 2000 and 2016 in all the three countries and was rated as ‘high’ in Mongolia (10.1%), ‘low’ in the Philippines (4.2%) and ‘moderate’ in Vietnam (6.0%) (Figure 1A).

The prevalence of overweight and obesity among children and adolescents aged 5−19 years has also increased, and absolute values are greater than for children aged < 5 years (Figure 1B). It was rated ‘high’ in Mongolia (increasing from 11.5% in 2000 to 17.8% in 2016) and the Philippines (increasing from 6.4% in 2000 to 12.8% in 2016) and ‘moderate’ in Vietnam (increasing from 2.1% in 2000 to 9.7% in 2016). In the Philippines, the prevalence of the overweight and obesity is higher among the wealthier groups compared to less wealthy groups. In Mongolia, the prevalence of overweight and obesity was comparable between income groups (Supplementary File 4).

### Key risk factors for overweight and obesity

There were differences between the countries in critical risk for overweight and obesity among children under 5 years of age (Table 1). In Mongolia, high birth weight was the only risk factor rated ‘poor’ (12.6%). Maternal overweight and obesity and breastfeeding practices were considered ‘moderate’. In the Philippines, maternal overweight and obesity (35.2%), low birthweight (14%), high birthweight (11.2%) and stunting (28.8%) were all rated ‘poor’. Breastfeeding practices were rated ‘moderate’. In Vietnam, no risk factors were rated ‘poor’. Maternal overweight and obesity, low birthweight, stunting and breastfeeding practices were all rated ‘moderate’.

Table 2 presents the risk factors for the overweight and obesity in children aged 5−19 years. In all the three countries, diet was a critical risk factor, with ‘poor’ (insufficient) consumption of fruits and vegetables in all countries, and frequent consumption of fast food in Mongolia and the Philippines. While consumption of sugary drinks was ‘moderate’ (20−40% of adolescents consuming at least one serve daily) in all the countries, the proportion of schools without clean water was high in the Philippines (53.1% of schools did not have access to at least basic drinking water). The proportion of children meeting physical activity recommendations (≥60 min/day) was rated ‘poor’ in all countries, while use of active transport and sedentary behaviours among school-aged children were rated ‘moderate’, in all the three countries. Lack of sleep was a risk factor rated as ‘poor’ in Vietnam (43% of children routinely had <8 h of sleep per night).

### Policies that influence overweight and obesity among children

While all the three countries have enacted policies to address aspects of overweight and obesity (Supplementary File 3), there are critical gaps (Table 3). No country has a comprehensive policy for addressing the childhood overweight and obesity up to 19 years of age. Population-wide nutrition strategies exist and include specific targets for children though there are gaps for school age children. The Mongolian National Programme on Nutrition 2016−2025 does not include overweight and obesity targets for children aged 5−11 years. The Philippine Plan of Action for Nutrition 2017−2022 prioritizes addressing overweight and obesity among the adults. In Vietnam, the National Strategy on Nutrition 2021−2030 does include priorities and targets for addressing child malnutrition in its all forms but is predominantly focussed on addressing undernutrition.

The Philippines is the only country to have introduced a tax on sugar-sweetened beverages (SSBs), though Vietnam and Mongolia are currently considering such policies. In the Philippines, marketing of unhealthy foods and beverages is restricted by a voluntary, industry-led pledge (though European research suggests that the effectiveness of such pledges at protecting children is limited (Landwehr and Hartmann, 2020). There are no policies restricting unhealthy food or beverage marketing in Mongolia or Vietnam. No country has a mandatory interpretive or warning FOPNL. The Philippines and Mongolia have respective FOPNLs. However, in the case of the Philippines, this does not identify less-healthy products, and in both countries, these are voluntary to implement. All countries have implemented legislation regulating the marketing of BMS. The legislation in Mongolia and the Philippines closely aligns with the international code on the marketing of breastmilk substitutes (Global Breastfeeding Collective, 2017). In Vietnam, the legislation is only moderately aligned.

There is some regulation of school nutrition environments in all the three countries. In Mongolia, highly processed foods, fast foods, SSBs, packaged snacks and other unhealthy foods are prohibited from sale in schools. In the Philippines, marketing of unhealthy foods and beverages in schools is prohibited, and restrictions on the sale of unhealthy foods and beverages in schools are recommended, though this is voluntary. In Vietnam, the school meals programme and the school milk programme lack targets relating to childhood overweight and obesity, and so available foods and beverages are not restricted to ‘healthier’ products.

### Policy options for prevention of overweight and obesity among children

A total of 91 interviews were conducted with key stakeholders to identify key barriers and concerns in relation to addressing the childhood overweight and obesity: 44 in Mongolia, 11 in the Philippines and 36 in Vietnam. Interviewees included representatives from local and national government departments, intergovernmental agencies, advocacy organizations, professional societies and academic specialists. Four key overarching themes were constructed from the interview summaries provided by in-country experts: (1) the absence of political prioritisation of childhood overweight and obesity, (2) limited support for existing policies, (3) a perceived need for increased community education and understanding and (4) the influence of the food and beverage industry.

1. *Absence of political prioritization to address childhood overweight and obesity*

Across all countries, interviews with key stakeholders revealed the perception that the prevalence of childhood overweight and obesity is not as high as in high-income countries. There was a perception that the prevalence of undernutrition among the young children is still the primary nutrition-related concern of governments. Where childhood overweight and obesity are recognized, it is only as a risk factor for NCDs. This is manifested in an absence of policies to address the childhood overweight and obesity in Mongolia, Vietnam and the Philippines.

[There are] *only… general policies on food and nutrition, there is no specific policy for childhood overweight/obesity. The content is… on proper nutrition in general, but it is not clear what to do for obese children. There is no professional guidance or instructions, that are specific to overweight/obesity*. **Vietnam—Government Agency**

Government leadership was recognized as playing an important role in addressing childhood overweight and obesity, in particular due to the legislative, financing, allocative (of resources), enforcement, monitoring and surveillance powers of government at a national level.

*But then I believe that the only way we can address the problem of obesity is to involve all sectors of the society; schools, community, and of course government. The government will play a very important role in addressing the problem of obesity.* **Philippines—Academic and medical society**

2. *Limited support for existing policies*

Where policies to address childhood overweight and obesity are implemented, they are often limited by the level of support they receive, specifically from government and legislative bodies. Interviewees largely discussed the importance of financial and resource support but also highlighted the symbolic role of support from government and government agencies. At a national level, this can be seen in an absence of resources and funds allocated to key policies:

*The funding for implementing intervention/program on childhood overweight is very limited, almost none, there is also a lack of coordination among ministries, and human training.* **Vietnam—Government Agency**

At a local level, where implementation is commonly realized, interviewees perceived that there is a disconnect between what is required of a policy and what can be feasibly accomplished. More specifically, existing policies may not be optimized for local contexts:

*According to regulations, the kitchen staff must have a health certificate and a certificate of training in food safety and hygiene. However, they are just contract workers, so their benefits (salary) are not guaranteed, making [it] easy to quit. It takes a lot of effort to train new employees.* **Vietnam—Government Agency**

This is likely a key driver of why it was seen as important to support monitoring of policies and programmes to ensure their adequate implementation, as well as their evaluation to ensure effectiveness:

*Most schools have physical education, which is a compulsory subject. However, in many schools, this is just a mere formality program and does not actually improve the child’s physical activity.* **Vietnam—UN Agency**

3. *Lack of community education and understanding*

Some key informants identified a lack of knowledge and understanding as a barrier to appropriate IYCF practices. More than this, stakeholders perceived that there was a lack of interest and attention from the community regarding the impacts of unhealthy lifestyle habits on health:

*Unable to issue a policy because people are indifferent and do not pay much attention to health issues such as [that] drinking a lot of sugary drinks may cause obesity and heart diseases.* **Vietnam—Government Agency**

A need for a synchronized education approach that targets consumers in multiple settings and at multiple life stages was perceived as important by some interview participants. Changing at-home diets through targeting family units was identified as a key strategy for improving population diets.

*“\First of all, for me, the parents have a big role at home. Although let’s say in school, it is taught that the children should eat delicious foods or nutritious foods, etc. etc. but at home, other foods are consumed.* **Philippines—Government agency**

4. *Influence of the food and beverage industry*

The absence of political prioritization of childhood overweight and obesity was also perceived to increase the ability of the food and beverage industry to influence nutrition policies, as the lobbying by the industry was less likely to be met with determined opposition. This lobbying has the potential to limit policy implementation and reduce the efficacy of policies that are implemented.

*The policy community cohesion in the opposite parties is clearly strong, including commercial beverage companies. They organized workshops, wrote to the government with evidence that showed sugary drink was not the cause of obesity in Vietnam… and imposing taxes on those products would cause harm to the economy.* **Vietnam—Government agency**

While education campaigns were identified as a key policy for addressing the childhood overweight and obesity, marketing of unhealthy food and beverages by the food and beverage industry is likely to counter the benefits of any educational campaigns. The quantity of marketing of unhealthy foods and beverages, particularly across social media sites, was noted as a concern by interview participants:

*…especially for online advertising on social networking sites. For example, the problem of advertising carbonated drinks, since people use many social networks, like Facebook, YouTube, which are out of the control of the government, thus we can’t control it, we can’t prevent it.* **Vietnam—Government agency**

Hence, while there is acknowledgement of the need for action to address childhood overweight and obesity, strategies to counter the actions of the food and beverage industry are also essential across Mongolia, the Philippines and Vietnam.

### Policy priorities for prevention of overweight and obesity among children

In all, 44 stakeholders in Mongolia and 52 stakeholders in the Philippines participated in validation workshops. The key policies identified in Mongolia and the Philippines are similar (Supplementary File 5). The key role of governance and the need for overarching strategies and nutrition councils was highlighted. Policies, aimed at improving the food environment, were identified as critical. These included the introduction of FOPNLs, restrictions on the marketing of unhealthy food and beverages, taxation on unhealthy food and beverages, and ensuring that school environments are supportive of healthy eating and physical activity through the development of school food and nutrition standards, nutrition literacy and physical education. The importance of urban environments, water, hygiene and sanitation systems, and social protection systems was also recognized. This included ensuring that urban areas were supportive of physical activity and transport, that potable drinking water was readily available, and that healthy and affordable food was available to all.

## DISCUSSION

This landscape analysis aimed to synthesis and describe the available evidence on the prevalence and trends in childhood overweight and obesity and related risk factors, and discuss the policy response to this, in Mongolia, Philippines and Vietnam. Across all three countries, the prevalence of overweight and obesity among children aged 5−19 years has climbed by more than 6%-points from 2000 to 2016. While the prevalence of overweight and obesity among this age group is higher in high-income countries in this region, the rate of increase is greater in the LMICs presented herein (Huse *et al.*, 2022). Unhealthy diets and insufficient physical activity were common risk factors in all three countries. Despite differing country contexts, increasingly obesogenic environments combined with a lack of sufficient policy implementation are contributing to the growth in childhood overweight and obesity. Similar barriers to addressing childhood overweight and obesity were identified.

Despite differences in degree of urbanisation, food production and diets, there are similarities across the countries in the risk factors for overweight and obesity in children. This confirms findings from previous studies which found associations with maternal nutrition and birthweight (Borja, 2013), breastfeeding (Nguyen *et al.*, 2021), diets (Ohly *et al.*, 2022) and physical activity (Trang *et al.*, 2012; Nguyen *et al.*, 2016). While key informants perceived that individual choice is a driver of overweight and obesity, the results of the landscape analyses suggest that children are living in increasingly obesogenic environments that reduce their opportunities to access healthy food and to be physically active.

There is an absence of best-practise policies including restrictions on unhealthy food and beverage marketing, FOPNL, and SSB taxation policies, particularly in Mongolia and Vietnam. There is strong evidence to show that national governments can play a vital role in preventing overweight and obesity in children (Brambila-Macias *et al.*, 2011; Mozaffarian *et al.*, 2018). It has been demonstrated that a suite of policy actions can drive transformative food system change and is more effective than isolated policy actions (Brambila-Macias *et al.*, 2011; Mozaffarian *et al.*, 2018). Despite this, while the Philippines has some key policies in place, implementation, monitoring and enforcement are frequently lacking. Interviews with key informants highlighted the barriers to policy implementation that include (i) an undefined problem and solutions, (ii) inadequate resource allocation and (iii) limited community understanding and awareness of overweight and obesity. Notably, the actions of the food and beverage industry were also identified as a key driver of both unhealthy food environments and inadequate policy implementation, and previous research notes the interest of these corporations in LMICs (Stuckler and Nestle, 2012; Moodie *et al.*, 2021).

In the absence of political leadership and robust policies, the food and beverage industry has expanded, increasing the sale and marketing of unhealthy products to children and lobbying governments to avoid restrictions (Baker and Friel, 2014, 2016). The activities of the BMS and complementary food industries have been identified as a key driver of poor breastfeeding rates in the Philippines (Baker *et al.*, 2021). In Manila, the Philippines and Ulaanbaatar, Mongolia, food and beverage advertisements were more common in and around schools and were almost always promoting unhealthy products (Kelly *et al.*, 2015). Online and digital marketing is also increasing, and frequently targets children (Bragg *et al.*, 2017; Tatlow-Golden and Boyland, 2021). The industry lobbied against sweetened beverage taxation (Onagan *et al.*, 2019) and school food environment guidelines (Reeve *et al.*, 2018) in the Philippines, highlighting the importance of protecting nutrition policy development from industry interference.

Introduction and implementation of a set of evidence-based policies could effectively improve diets and physical activity and halt the rise in overweight and obesity among children in East Asia. These policies include introduction of FOPNLs, restrictions on the marketing of unhealthy food and beverages, taxation on unhealthy food and beverages, and ensuring that school environments are supportive of healthy eating and physical activity. Policies need to be government led, mandatory and carefully monitored to ensure full implementation. To support these policies, monitoring and surveillance systems that routinely and comprehensively collect data should be implemented to facilitate effective policy monitoring and evaluation. However, an effective policy response is unlikely to be realized while the power of the food and beverage industry remains unchecked (Gilmore *et al.*, 2023). The financial and symbolic power available to these corporations gives them leverage over policy processes, and subsequently empowers these corporations to act and argue against the implementation of the most effective food and nutrition policies (Wood *et al.*, 2021). As such, any policy response to the childhood overweight and obesity should be supported by policies aimed at reducing the influence of the food and beverage industry, including conflict of interest and mandatory declaration requirements (Mialon *et al.*, 2021). Such a response will require a strong and united coalition of civil society, medical, public health and academic actors (Lacy-Nichols *et al.*, 2022).

Also of note is that interviewees identified limited community understanding and awareness of overweight and obesity as a barrier to addressing the childhood overweight and obesity. This is counter to the literature which suggests that environmental factors are a key driver of overweight and obesity (Poston and Foreyt, 1999). In the Philippines, the food and beverage industry has been reported to capitalize on perceptions that undernutrition remains the dominant health concern (Huse *et al.*, 2023), and this may be driving the perceived need for educational policies here and elsewhere. It will be important that LMICs in the Western Pacific region and beyond are supported to enact a wide range of policies, including policies that were included in the recommendations synthesized through this study (Poston and Foreyt, 1999).

An important strength of the research was the use of a structured protocol that could be applied across multiple contexts and combined quantitative and qualitative research methods. A further strength was the inclusion of underlying environmental drivers of overweight and obesity as well as immediate risk factors so shifting the focus from individual responsibility to addressing the environments within which children live. The study successfully linked data on the prevalence of, trends in, and risk factors for childhood overweight and obesity to policy actions, and then brought together policymakers and key stakeholders from across sectors to identify priority actions. Finally, data collection was undertaken by national researchers with in-depth knowledge of the country and the environment, adding a layer of contextual knowledge to the exercise.

This research had limitations. No primary data were collected, and the analyses were dependent on secondary sources, each with their own limitations. For example, the GSHS is limited to a specific age group of children and is infrequently collected (World Health Organization., 2022b), and the UNICEF/WHO/World Bank Joint Malnutrition Estimates (United Nations Children’s Fund *et al.*, 2012) and NCD RisC database (NCD Risk Factor Collaboration, 2017) draw on modelled estimates. However, the selected data sources represent the best options available for Mongolia, the Philippines and Vietnam, where national surveys may not capture all data of interest. The GSHS provides data on a range of indicators not commonly included in other surveys, including children’s SSB, fast food and fruit and vegetable consumption, excessive sedentary behaviour and active transport to school (World Health Organization., 2022b). Furthermore, as data for each country was collected by a different researcher it is possible that their different perspectives may have coloured the data collection process. Data collection was conducted with a view to informing policy rather than an academic manuscript. As such, in-depth research methods were not adopted. Finally, data collection was conducted during the global COVID-19 pandemic, and this limited the quantity and variety of interviews that could be conducted.

The UNICEF landscape analysis tool was successfully used to describe childhood overweight and obesity, the related risk factors and policy responses in Mongolia, the Philippines and Vietnam, and to identify priority actions. The three countries of Mongolia, the Philippines and Vietnam are all facing an increasing burden of overweight and obesity among children due to a range of critical risk factors. Despite the differing contexts, similar environmental factors are driving the rise in risk and prevalence of overweight and obesity. Strong political leadership and prioritization of effective legislation and policy action are urgently required to halt the rise in child overweight and obesity in the Western Pacific region.

## Supplementary Material

daad187_suppl_Supplementary_Files_1-5
