# Supplementary material for: Prevention of childhood overweight and obesity in Mongolia, the Philippines and Vietnam: identifying priority actions
Source: Health Promot Int. 2023 Dec 29;38(6):daad187. doi: 10.1093/heapro/daad187 (PMC10756053; doi:10.1093/heapro/daad187)
Supplement: daad187_suppl_Supplementary_Files_1-5 [file daad187_suppl_supplementary_files_1-5.docx]

**Supplementary File 1: Data sources included in the landscape analysis**

- The UNICEF/WHO/World Bank Joint Malnutrition Estimates have been described in detail elsewhere^(1)^. UNICEF and WHO receive and review survey data from academic, grey and government sources on a regular basis. Based on these data, UNICEF and WHO develop and maintain a dataset of national child malnutrition prevalence estimates for children aged <5 years for all countries or territories with available survey data.
- The NCD-RisC database has also been described in detail elsewhere^(2)^. NCD-RisC is a worldwide network of health researchers that provides data on NCD risk factors, including overweight and obesity. Data from population surveys are combined through pooling analyses to produce estimates of NCD risk factors (including BMI)^(2)^.
- The GSHS is a school-based survey conducted amongst students aged 13-17 years using a standardized scientific sample selection process, common school-based methodology and self-administered, country-specific questionnaire^(3)^. The UNICEF IYCF Database provides data on a range of IYCF practices^(4)^. Data for these indicators are collected through household surveys such as DHS, MICS, and other national nutrition surveys.
- UNICEF and WHO have compiled estimates of the proportion of children born with a low birthweight (<2.5kg) from 2000–2015 for 147 countries^(5)^. Data are obtained through systematic searches of National Statistical Office and Ministry of Health websites, from websites of the household survey programmes of Multiple Indicator Cluster Surveys and Demographic and Health Surveys, and from data gathered during an extensive country consultation.
- The WHO Global Health Observatory is the WHO’s primary health statistics data repository^(6)^. It collates data from civil registration authorities, population censuses, household surveys, administration reporting systems, surveillance systems and facility reporting systems from member states to provide access to over 1000 indicators on priority health topics.
- The WHO/UNICEF Joint Monitoring Programme for Water Supply, Sanitation and Hygiene^(7)^ includes estimates for access to basic drinking water in schools and households. Estimates are based on data collected through national Education Management Information Systems, censuses, and surveys, as well as secondary sources in the absence of primary data.

**Supplementary File 2A: Risk factors for the development of overweight-for-height amongst children aged <5 years**

| **Risk factor** | **Indicator** | **Thresholds** |
| --- | --- | --- |
| ***Pre-natal risk factors*** | | |
| Maternal obesity | Proportion of women of child-bearing age with body mass index (BMI) ≥30kg/m^2^ | Good: <10%  Moderate: 10% to <25%  Poor: ≥25% |
| Maternal tobacco smoking | Proportion of women of child-bearing age who smoke tobacco | Good: <5%  Moderate: 5% to <15%  Poor: ≥15% |
| ***Post-natal risk factors*** | | |
| Low birthweight | Proportion of children born with birthweight <2.5kg | Good: <5%  Moderate: 5% to <10%  Poor: ≥10% |
| High birthweight | Proportion of children born with birthweight >4.0kg | Good: <5%  Moderate: 5% to <10%  Poor: ≥10% |
| ***Stunting*** | | |
| Stunting amongst infants and young children | Proportion of children with weight-for-height ≤2 standard deviations below the median of the WHO Child Growth Standards reference population ^(8)^. | Good: <10%  Moderate: 10% to <20%  Poor: ≥20% |
| ***Infant and young child feeding*** | | |
| Early initiation of breastfeeding | The proportion of children who were initiated to breastfeeding within one hour after birth | Good: >70%  Moderate: 30% to 70%  Poor: <30% |
| Exclusive breastfeeding | The proportion of children who were exclusively breastfed for the first 5 months of life | Good: >70%  Moderate: 30% to 70%  Poor: <30% |

**Supplementary File 2B: Risk factors for the development of overweight and obesity amongst children aged 5-19 years**

| **Risk factor** | **Indicator** | **Thresholds** |
| --- | --- | --- |
| ***Dietary risk factors*** | | |
| Sugar-sweetened beverage (SSB) consumption | Proportion of children consuming SSBs at least once per day | Good: <20%  Moderate: 20% to 40%  Poor: ≥40% |
| Fast food consumption | Proportion of children consuming fast food at least once per week | Good: <20%  Moderate: 20% to 40%  Poor: ≥40% |
| Fruit or vegetable consumption | Proportion of children consuming <3 serves of fruit or vegetables per day | Good: <40%  Moderate: 40% to 60%  Poor: ≥60% |
| Fruit and vegetable consumption | Proportion of children consuming <5 serves of fruit and vegetables per day | Good: <40%  Moderate: 40% to 60%  Poor: ≥60% |
| Access to safe drinking water in schools | Proportion of schools without access to at least ‘basic’ drinking water. | Good: <10%  Moderate: 10% to 50%  Poor: >40% |
| Access to safe drinking water in households | Proportion of households without access to at least ‘basic’ drinking water. | Good: <10%  Moderate: 10% to 50%  Poor: >40% |
| ***Physical activity risk factors*** | | |
| Insufficient physical activity | Proportion of children engaging in ≤60 minutes of moderate to vigorous physical activity per day | Good: <40%  Moderate: 40% to 70%  Poor: >70% |
| Excessive sedentary behaviour | Proportion of children engaging in ≥3 hours of screen time per day | Good: <40%  Moderate: 40% to 70%  Poor: >70% |
| Use of active transport to travel to school | Proportion of children who do not walk or ride a bicycle to school | Good: <40%  Moderate: 40% to 70%  Poor: >70% |
| Insufficient sleep | Proportion of children who have ≤8 hours of sleep per night | Good: <10%  Moderate: 10% to 20%  Poor: >20% |

Supplementary File 3A: Environments and policies that determine the risk factors for overweight and obesity in Mongolia

| **Key questions** | **Information** |
| --- | --- |
| ***National Policy*** | |
| Does the national government have a written policy addressing obesity? Does this specifically include targets (infants, under 5s, and children)? | Yes, national target consistent with WHO targets |
| Do any of the policy statements discuss double burden or double-duty actions to tackle obesity? | Yes, Healthy Eating for All policy |
| ***Food Based Dietary Guidelines and Nutrient Profiling Schemes*** | |
| Are there government-endorsed national Food Based Dietary Guidelines (FBDGs)? | Yes, dietary guideline and food guide Ger for healthy eating |
| Is there a government-endorsed nutrient profiling scheme (NPS)? | Yes, approved by Health minister order #215 of 2018 adopted from Western pacific regional scheme  Implementation of nutrient profiling scheme is voluntary. |
| Are there specific subsidies, taxes, or levies on foods or beverages linked explicitly to the government endorsed FBDGs or NPS? | No, except special taxes for alcoholic beverages and tobacco. |
| Are there any examples of government procurement policies linked specifically to FBDGs or NPS? | No. General quality standards requirements, not nutrition-based |
| Are there any local or national controls on commercial catering services (including fast-food chains) linked specifically to FBDGs or NPS? | Yes, only in school environment.  Health minister approved the list of foods (foods high saturated fat, trans fat, sugar or salt, HFSS) that should not be sold in the school environment within 150 meters (order #369, 2020). |
| ***Food Labelling and Marketing*** | |
| Is there a government-endorsed front-of-pack and/or menu nutrition labelling scheme which signals foods high in fats or sugars? | Yes. Pre-packaged food labelling is mandatory, with side of pack ingredients and nutrient lists. Front of pack labelling is voluntary. Nutrient and health claims follow Codex recommendations.  Yes. In 2019, the Head of Mongolian agency for Standard and Metrology approved amended version of Mongolian national standard “MNS 4946:2019, Requirements for Food service establishment”. The amended standard has special provisions about placing nutrition fact information (calories, salt and sugar contents) on the menu. |
| Government advisory body for food security + nutrition policy? | Yes, National food security council includes Mongolian food producers’ association. |
| Monitoring food distribution by companies? | GASI is the Government authority responsible for regulatory monitoring on implementation of policies and strategies.  GASI is responsible for regulatory monitoring of food production, trade, and commercial catering services and BMS and complementary food distribution. |
| ***Media and Food Advertising*** | |
| Are there policies to control what types of food and beverages are being promoted? | No marketing regulation for HFSS foods. General rules on truthful advertising. TV marketing time bound restriction for alcoholic beverages and tobacco products is in place.  Authority for Fair Competition and Consumer Protection of Mongolia is Government regulatory agency in charge for enforces Competition Law, Consumer Protection Law and Advertisement Law. |
| Are chain restaurants/fast-food stores offering healthy versions of popular products? | No, there are no standards for the ingredients or nutritional value of dishes for public catering services. Therefore, no policies or measures have been implemented to classify them as healthy or unhealthy or different price policies. |
| ***Food reformulation, taxation, subsidy, and research*** | |
| Are there government-set targets for reducing salt, fat, or sugar for health purposes? | Yes. The National Salt Reduction Strategy aims to reduce salt consumption by 30 percent. The first goal is aimed to create a legal environment to support the import, production, trade, service, and consumption of low-salt foods. In the framework of the strategy, MOH and MOFALI implementing a 2-year campaign to promote low salt food production based on volunteer participation of the food producers. |
| Is there a policy to subsidise or encourage home-produced foods which meet nutrition criteria? | In some ways, agriculture production is linking with FBDGs. Mongolian Government is implementing the National programmes on “Vegetables” and “Fruit and berries” for 2018-2022. These programme aims to support domestic production of vegetables, fruits and berries. Currently, the Mongolian government provides cash incentives only to wheat farmers (individuals and enterprisers). However, support is provided to vegetable, fruit and berry farmers through non-cash incentives and soft loans. |
| Is there a policy to subsidise or support food chain processes specifically linked to government endorsed FBDGs or NPS criteria? | Not specific. In order to create a food supply chain for public schools and preschool care centres (kindergartens), local authorities established a state-owned local enterprise. These enterprises are responsible for safe, nutritious food supply for nominated community territories. |
| Is there a policy for restricting imports of foods that fail to meet specific nutritional criteria? | No policy for restricting imports of foods which fail to meet specific nutrition criteria. |
| Is there a policy to support research or development that is linked to government endorsed FBDGs or NPS criteria? | Support for small and medium scale farmers through noncash incentives and soft loans. |
| ***Environmental and cultural factors*** | |
| Are local water supplies believed to be safe to drink? Is water widely available? | 86.9% household has access to safe drinking water and 56% of schools providing students with free drinking water that meets the minimum standard requirements. But sugary drinks consumption is common, especially in urban areas. |
| Are there specific cultural norms that reduce the opportunities to take physical activity? | Physical activity and sports are encouraged. Mongolians have traditionally physically active lifestyle. Due to urbanization, physically active lifestyle is shifted to western lifestyle. Use of motorbike or cars are increasing rapidly among urban and rural residents, even in herders.  There is no cultural inhibition for physical activity or sports. |
| Does the prevailing climate or terrain affect dietary behaviour or physical activity? | Yes, the climate is strongly continental, with long, frigid winters and short, warm summers. The long, frigid winter limits children’s active play and time spent outdoor.  Mongolia has been one of the most polluted countries in the last two decades. The most polluted air in Mongolia is found in Ulaanbaatar, where 46% of the country’s population resides. Due to long lasting cold winter and air pollution, urban and Ulaanbaatar city residents are living in limited conditions to be physically active. |
| Are there policies at national or city level to provide safe cycling and walking routes? | No cycle lanes, except Ulaanbaatar. Few urban parks have bicycle lanes. Although in Concept of the Mongolian Sustainable Development – 2030 and the Ulaanbaatar city Master Plan has objectives to support active transports, the length of bicycle lanes is insufficient. |
| Are there policies at national or city level to reduce car use? | No direct policy or measures to decrease car use. Anti-pollution measures are mostly oriented to decrease use of raw coal in Ulaanbaatar. The concept of the general development plan of Ulaanbaatar city - 2040 and the action plan of the Mayor of Ulaanbaatar for 2020-2024 has no special clauses to reduce car use. However, these documents have special directions to develop city public transport. |
| Is there evidence of a code of conduct for media on reporting on obesity and avoiding stigma? | No evidence of anti-fat bias, but some emphasis on women’s ‘slim’ shape. |
| Are there cultural preferences for particular foods or cooking practices? | Yes: Mongolia is well known for its nomadic traditions. The nomadic way of lifestyle is still practiced today. Due to nomadic tradition, Mongolian cuisine predominantly consists of meat, dairy products, and animal fats. Food consumption is differing by urban and rural settings. Rural people consuming relatively high amount of meat, milk and dairies, wheat flour and cereals. Urban Mongolians consumption rate of vegetables, fruits and eggs are relatively higher than rural residents. |
| Are there cultural reasons why excess weight might be encouraged? | No cultural reason or beliefs to encourage excess weight, but in some folk tale’s poverty is described with thinness.  In some way, an overweight baby is encouraged by the community. People encourage young children with round cheeks. |
| Are there specific rules about feasting or fasting which may encourage weight gain? | No specific rules. |

Supplementary File 3B: Environments and policies that determine the risk factors for childhood overweight and obesity Mongolia

| **Key questions** | **Information** |
| --- | --- |
| **Level of National Support** | |
| Is there visible support from leadership for action on childhood obesity? | Yes, support for WHO and UNICEF initiatives. Except for the MOH, there is no specific ministries that recognize childhood obesity and links to undernutrition. |
| Is there an obesity policy coordination platform for national and local government/NGO collaboration? | No obesity body and/or nutrition council.  National Food Security Council is established in 2013 (includes Food producers association). |
| Are there city-led or local authority-led strategies and policies to tackle child obesity? | Yes, local authorities are responsible for coordination of implementation of the public health programmes (nutrition) and strategies at the local level.  The local governor has the authority to approve sub-programs and action plans for their implementation. |
| Is surveillance to monitor child obesity, or diet and nutrition, conducted? | Weights are monitored of children under 5 years of age. In November 2019 the Minister of Health and Minister of Education, Science and Sport endorsed the “Regulation on surveillance and recording of body weight and height of school children”. This regulation should be implemented by public and private schools (secondary and high), within the framework of the physical education curriculum. Physical education teachers, school doctors and education supervisors are responsible for accurate measuring and reporting of the weight and height of school students aged 6-18 years.  Dietary data for school children is limited except the Global School-Based Student Health Survey (GSHS), conducted in 2013. |
| Does the government have obesity (and undernutrition) targets? | Yes, National programme on Nutrition has special targets for child malnutrition (overweight, obesity, stunting, and wasting) in line with WHO/UNICEF targets. |
| Is there monitoring of the media narrative on child weight? | No media monitoring. The Authority for Fair Competition and Consumer Protection (AFCCP) is the government regulatory agency in charge of enforcing the Consumer Protection Law and Advertisement Law. While AFCCP is responsible for regulatory monitoring of the media, there are no official reports on protection of children from harmful media advertisements related with foods and drinks.  Health and nutrition experts’ advice on child feeding and nutrition, information related to the prevalence of overweight and obesity in children can be found on public websites, newspapers, and magazines. Unfortunately, there is no information related to the responsibilities of the media sector and food producers for the prevention of childhood obesity. |
| Is there good public access to government data on obesity-related policy-making? | Limited documentation that is easily accessed. |
| Is the government funding nutrition education promotion programs? | Yes, for adolescents (in curriculum), and pre- and in-service training for health workers. |
| Is the government funding nutrition interventions for children? | Yes, targeted for promotion of healthy growth in children under 5 years of age (growth monitoring, supplementation of high dose vitamin A, multi-micronutrient supplements for pregnant and lactating women, young children 6–23 months of age).  Except school lunch programme, there are no specific nutrition interventions targeted for older children. |
| Is the government funding obesity prevention research? | No, but legally an individual researcher or research team (institute) can develop a scientific project or survey proposal on a topic of interest and submit it to the Science and Technology Foundation for approval in accordance with the relevant procedures. |
| Is there a standard procedure to assess the impact of food-related policies on health and obesity? | No. Mongolia has no standard procedure or protocol to assess the impact of food related policies on health and obesity. |
| Are there any monitoring reports of HIAs? | No. |
| Are there any monitoring reports of food company activities on HFSS food promotion? | Yes. GASI is looking at BMS violations. |
| ***Health System: During pregnancy*** | |
| Are adolescent and maternal nutrition programmes provided? | Yes. The national programme on nutrition has specific objectives to improve maternal and child nutrition status. Within the framework of this objective, planned to implement activities to promote healthy diet. Among these activities no specific provisions to tackle maternal obesity.  Attendance of antenatal care is universal (88.5%). Antenatal care includes healthy eating classes. |
| Do women receive antenatal monitoring to prevent excessive weight gain and maternal diabetes? | Yes, when attending antenatal clinics. But there are no specific actions for prevention of excessive weight gain and gestational diabetes. |
| Is there provision for prenatal counselling and care? | Yes, if attending antenatal clinics. In accordance with Health Minister order 338 (October 20, 2014), under the ANC services pregnant women receive counselling on diet, harmful addiction to alcohol and smoking, use of supplements, and risk of anaemia. There are no specific provisions counselling on prevention of malnutrition (both underweight and/or overweight) |
| Are protein and/or energy supplements provided to pregnant women? | No. Protein and energy supplementation is not provided for pregnant women.  However, free of charge multiple micronutrient supplementation for pregnant women is provided. |
| ***Health and Social Support systems: During infancy and young childhood*** | |
| Are there programmes and practices to promote breastfeeding? | Breastfeeding promotion is enforced by Mongolian Law on Food products intended for infants and toddlers.  Breastfeeding promotion is addressed in the following 2 national programmes endorsed by the Mongolian Government. 1) National programme on Nutrition (Objective 2. Improve maternal and child nutrition status, 2) National programme on the promotion of maternal, infants and toddler's healthy diet. |
| Are hospitals following the recommendations of the Baby-Friendly Hospitals Initiative? | Yes. Health Minister endorsed order A-29 of June 13, 2019 on "Guidance for implementation of 10 steps for successful breastfeeding". The purpose of this guidance is to introduce the “Ten Steps for breastfeeding promotion" into the activities of health facilities providing maternal and child health care services.  Updated “Accreditation criteria for health care facilities providing professional medical care services” was approved by Health Minister order #A-554 on December 6, 2019. Breastfeeding promotion 10 steps are included into part 8 (Obstetrics, **gynaecology** and newborn care) of Chapter 3. Indicators for evaluation of health care service quality, safety, and technology of this accreditation criteria are available. |
| Do women have a right to maternity leave? | Yes, through the government social insurance service. |
| Is the International Code on the Marketing of Breast-milk Substitutes implemented by national legislation? | In 2017, Mongolia adopted new legislation on marketing of BMS. The recent legislation is a distinct improvement over its predecessor from 2005. It introduces many elements that were missing in the 2005 law. However, not all the important elements of the Code are incorporated into the new law. The Departments of health, education, and culture inspection of the GASI is responsible for regulatory monitoring of law enforcement in the health sector. |
| Is there screening to monitor infants and young children for overweight or other malnutrition? | Yes. Under age 5, focus on undernutrition.  In November 2019 the Minister of Health and Minister of Education, Science and Sport endorsed the “Regulation on surveillance and recording of body weight and height of school children”. In accordance with this regulation, school children’s body weight and height shall be measured twice a year and reported. Due to the COVID–19 pandemic, implementation of this regulation has been postponed. |
| Are there policies or programmes to support families' access to healthy foods? | No specific policy or programs to support family access to healthy foods.  Since 2013, Mongolia has been implementing a food voucher programme for households in need of social support and assistance specified in the Law on Social Welfare. The following 10 types of food products are provided to the target group household members by the “food voucher” from selected shops. These include: 1. Meat and by-products, (non-imported), 2. Milk and dairy products (non-imported), 3. Flour and bakery products (non-imported), 4. Butter, 5. Sugar, 6. Potatoes and vegetables, 7. Rice, 8. Vegetable oil, 9. Eggs and 10. Fruits and berries. |
| Are there referral routes for infants and young children at higher risk of overweight or obesity? | Currently, there are no specific guidelines for the screening and referral routes for children and adolescents with overweight and obesity in Mongolia. |
| Do primary health-care workers receive training in providing nutrition counselling? | Yes. General practitioner and nurses are trained in infant and young child feeding (IYCF). |
| Do new parents have access to parenting courses? | No official counselling service on parenting. |
| ***Education System*** | |
| Are there nutritional and physical activity standards for young children in child-care settings? Is there national guidance for screen-time and sleep-in child-care settings? | No standards for diet, physical activity and screen-time for child-care settings.  However, recommended daily energy intake and essential nutrients (protein, fat, carbohydrates) of kindergarten and school children and the reference intake of food products are approved by joint order No A/166, A/559, 222 of the Ministers of Education and Science, Health, and Finance (Amendment 3, December 02, 2020). Public and private childcare settings are responsible for implementing this joint order. |
| Are there nutrition standards for meals provided in schools? | Currently, there are no standards for meals (snack and/or lunch) provided in schools. However, recommended daily energy intake and essential nutrients (protein, fat, carbohydrates) of school children and the reference intake of food products are approved by joint order No A/166, A/559, 222 of Ministers of Education and Science, Health, and Finance (Amendment 3, December 02, 2020). Public and private secondary schools are responsible for implementing the joint order. |
| Are there standards for children’s physical activity? | No standards for children's physical activity. |
| Are there standards for sedentary time, or screen time, for children at school? | No standards for amount of sedentary time, screen time for children at school. |
| Is nutrition education included on the school curriculum? | Yes, secondary education core curriculum was approved by Minister of Education, Culture, Science and Sports order A/491 of August 1, 2019. The core curriculum includes health which covers nutrition and physical activity. This includes healthy eating and basic information on prevention from malnutrition. |
| Are there policies to ensure access to safe drinking water in schools and sport facilities? | Yes, in some schools. In 2019, 56% of secondary schools provided students with free drinking water that met requirements. |
| Are there policies to encourage active travel (walking and cycling) to and from school? | No |
| Are there policies to give access to school and municipal sport and play facilities outside of school hours? | No |
| Are there nutrition standards for other foods sold in schools, e.g., in vending machines? | Currently, there are no standards for snacks provided in schools. However, recommended daily energy intake and essential nutrients (protein, fat, carbohydrates) of school children and the reference intake of food products are approved by joint order of the Ministers of Education and Science, Health, and Finance. |
| Are there school-based fitness monitoring programmes? | Yes. In order to educate the population of Mongolia about healthy lifestyles and behaviour, and to create a new generation with physical and mental endurance to overcome any obstacles, the President of Mongolia endorsed Decree 53, March 26, 2010, to conduct the physical development and fitness test.  The Government of Mongolia is instructed to conduct an annual assessment in April and undertake necessary measures based on the results of physical development and fitness tests. The purpose of the Presidential Decree is to engage children and youth with physical education and sports, to create healthy and physically fit new generations. |

Supplementary File 3C: Environments and policies that determine the risk factors for overweight and obesity in the Philippines

| **Key questions** | **Information** |
| --- | --- |
| ***National Policy*** | |
| Does the national government have a written policy addressing obesity? Does this specifically include childhood obesity? Do any of the policy statements include targets (infants, under 5s, and children)? | No specific policies for overweight and obesity but there are targets for overweight reduction among children < 5, 6-10 years old, adolescents, and adults in Philippines Plan of Action for Nutrition (PPAN) 2017-2022 (NNC, 2017). |
| Do any of the policy statements discuss double burden or double-duty actions to tackle obesity as part of a wider malnutrition/hunger strategy? | Yes. Obesity/overweight is included in the Conceptual framework of malnutrition in PPAN 2017-2022 (NNC, 2017). |
| Is there a government-led platform, forum, committee, or other body which assists in food policy development? | Yes. The Interagency Task Force on Zero Hunger, the National Nutrition Council (NNC), the Department of Agriculture (DA), and the Food and Drug Administration (FDA). |
| ***Food Based Dietary Guidelines and Nutrient Profiling Schemes*** | |
| Are there government-endorsed national Food Based Dietary Guidelines (FBDGs) and do these include specific recommendations for children e.g. infants, young children, or adolescents? | Yes. The 2012 Nutritional Guidelines for Filipino, Daily Nutritional Guide Pyramid, and the healthy food plate for Filipinos (Pinggang Pinoy) (DOST-FNRI, n.d.). |
| Is there a government-endorsed nutrient profiling scheme (NPS), e.g., used to restrict marketing foods to children or to classify foods for front-of-pack labelling signals? | In progress. |
| Are there specific subsidies, taxes, or levies on foods or beverages linked explicitly to the government endorsed FBDGs or NPS? | Yes. Republic Act (RA) 10963 or the “Tax Reform for Acceleration and Inclusion (TRAIN) Law”. Section 47 |
| Are there any examples of government procurement policies linked specifically to FBDGs or NPS? | Yes. For schools and DepEd offices: DepEd Order No. 13 s. 2017: Policy and Guidelines on Healthy Food and Beverage Choices in Schools and DepEd Offices. |
| Are there any local or national controls on commercial catering services (including fast food chains) linked specifically to FBDGs or NPS? e.g. restrictions on allowing fast food stores near schools? | Yes. DepEd Order No. 13, s. 2017: Policy and Guidelines on Healthy Food and Beverage Choices in Schools and DepEd Offices. There are also several local government units that have local policies on catering services. |
| ***Food Labelling and Marketing*** | |
| Is there a government-endorsed front-of-pack nutrition labelling scheme which signals foods high in fats or sugars? Menus: are there policies to require food service operators to show nutritional information on their menus? | There is no available government-endorsed front of pack nutrition labelling scheme which identifies foods high in fats or sugars. There is a mandatory nutrient declaration set by the government (Department of Health, Administrative Order 2014-0030). |
| Is there evidence of company distribution policies undermining healthy nutrition? This includes BMS Code violations, but also other promotions that undermine health – e.g. soft drinks companies assisting small distribution businesses, or offering mobile distribution equipment or services in low-income areas, or where there are few stores? | Yes. Lists of companies which have violations under the Philippine Milk Code (BMS Code Violation) and Food Fortification Law are available and can be requested from the FDA or through foi.gov.ph. |
| ***Media and Food Advertising*** | |
| Are there policies to control what types of food and beverages are being promoted: on TV? in the street? on digital media? | There is currently no Executive Order that regulates the marketing of unhealthy foods high in fats, sugars and/or salt. Advertising in the Philippines is industry-regulated and led by the Ad Standards Council (ASC). The ASC screens ads of products covered by the Milk Code/ Executive Order 51 (infant formula, complementary foods, teats, and feeding bottles), over-the-counter drugs, food/dietary supplements, alcoholic beverages, and airlines and other carriers, for truth and fairness (ASC, 2016).   The 2014 "Philippine Responsible Advertising to Children (RAC) Pledge" is signed by 12 large food and beverage manufacturers in the Philippines. It requires that product advertisements (TV and print media) directed to children below 12 years old should meet the nutritional criteria based on the dietary guidelines for children developed by the industry. Advertising in primary schools will only be conducted with the school’s consent, for educational purposes only (Philippine Food and Beverage Alliance, 2010). |
| Is there evidence of a code of conduct for media companies, or for journalists, on reporting on obesity and avoiding stigma or victim-blaming? | None. |
| Are chain restaurants/fast food stores offering healthy versions of popular products (especially those for children)? | Some fast-food restaurants offer popular food items and make them "healthier" by adding vegetables to them (Adlaon, 2013). |
| ***Food reformulation, taxation, subsidy, and research*** | |
| Food composition/reformulation - Are there government-set targets for reducing salt, fat, or sugar for health purposes? | There are no current policies regarding food reformulation. The NNC is advocating for this (NNC, 2017) and the DOST-FNRI has offered to assist manufacturers and food chains with product reformulation to make them healthier (Martina, 2017). Policy makers are proposing a bill for a Trans Fat Free Philippines (“An Act to Protect Filipinos from the Harmful Effects of Trans-fatty Acids (TFA)).” |
| Is there a policy to subsidise or encourage (e.g. through market support or tax relief) home-produced foods which meet nutrition criteria, e.g. government-endorsed FBDGs or NPS? | There are no current policies which subsidise home-produced foods. During COVID-19, the Department of Agriculture provides free assorted seeds and planting materials for an array of vegetables to households nationwide (DA Communications Group, 2020). |
| Is there a policy to subsidise or support food chain processes (transport, warehousing, chilling, freezing) specifically linked to government endorsed FBDGs or NPS criteria? | There is no specific policy to subsidise or support food chain processes specifically linked to FBDGs or NPS criteria. The Food and Agriculture Organization of the United Nations (FAO), in partnership with the Government of the Philippines, developed a relevant Country Programming Framework for 2018-2024 (FAO, 2018). |
| Is there a policy for restricting imports of foods that fail to meet specific nutritional criteria, e.g., using FBDGs or NPS classification? | Yes. The Implementing Rules and Regulations of RA No. 8976; “An Act Establishing the Philippines Food Fortification Program and for Other Purposes”. |
| Is there a policy to support research or outreach training and development that specifically links to government endorsed FBDGs or NPS criteria? | There is no current policy which specifically supports research and development that links to government endorsed FBDGs. |
| ***Environmental and cultural factors*** | |
| Are local water supplies believed to be safe to drink? Is water widely available? | Yes. 94% of the Philippine population is using at least basic drinking water services in 2017 (UNDP Water Governance Facility, n.d.). |
| Are there specific cultural norms that reduce the opportunities to take physical activity? Or increase the need for sedentary behaviour? | No data available. |
| Does the prevailing climate affect dietary behaviour or physical activity? Does the natural terrain affect physical activity? | No data available. |
| Are there policies at national or city level to provide safe cycling and walking routes? | Yes. The DILG Memorandum Circular 2020-100 or the “Guidelines for the Establishment of a Network of Cycling Lanes and Walking Paths to Support People's Mobility” specifies this. |
| Are there policies at national or city level to reduce car use? This includes policies to improve air pollution as well as encourage outdoor activity. | No data available. |
| Is there evidence of a code of conduct for media companies, or for journalists, on reporting on obesity and avoiding stigma and victim blaming? | None. |
| Are there cultural preferences for particular foods or cooking practices? | No data available. |
| Are there cultural reasons why fatness might be encouraged? Are these changing? Are there gender differences? | No data available. |
| Are there specific rules about feasting or fasting which may encourage weight gain? | No data available. |

Supplementary File 3D: Environments and policies that determine the risk factors for childhood overweight and obesity in the Philippines

| **Key questions** | **Information** |
| --- | --- |
| ***Level of National Support*** | |
| Is there visible support from the president/prime minister/cabinet office for action on childhood obesity? | Limited. The "Health for All Agenda 2016-2022" includes interventions towards cancer, diabetes, heart disease, and their risk factors – obesity, smoking, diet, sedentary lifestyle, and malnutrition. However, the identified risk factors pertain more to adults than children (DOH, 2016).  The national government also supports to the Philippine Plan of Action for Nutrition (PPAN) 2017-2022. |
| Is there an obesity policy coordination platform for national and local government collaboration? or for government and NGO collaboration? | Yes. NNC is currently organizing an inter-agency multi-sectoral group to lead actions to address overweight and obesity (Philippine Obesity Task Force). The Philippine Association for the Study of Overweight and Obesity (PASOO), a private organization, is a leader in the prevention and control of obesity and its complications through education, research & advocacy. |
| Are there city-led or local authority-led strategies and policies to tackle child obesity? | Yes. Several local government units have local policies and programs which aim to reduce risks of childhood obesity. |
| Does the government (or other independent organisation) conduct surveillance to monitor child obesity levels, and report the results? or monitor diet and nutrition intakes? | Yes. DOST-FNRI conducts a National Nutrition Survey every 5 years and an updating survey every 3 years where anthropometric measurements are collected, and nutritional status is reported. |
| Does the government have obesity (and undernutrition) targets? Are they time-limited? | Yes. The PPAN 2017-2022 includes a target to have no increase in overweight prevalence among children and to reduce overweight prevalence among adolescents and adults. |
| What common assumptions are made about childhood obesity? How much are parents assumed to be responsible? How much are commercial activities held responsible? Is the government urged to do more? | No data available. |
| Is there good public access to government information and the evidence used for obesity-related policy-making? | Yes. The Freedom of Information website (<https://www.foi.gov.ph/>) provides access to government information, virtue of EO No. 2, s. 2016. |
| Is the government funding nutrition education promotion programs? | Yes, the government funds the nutrition education programs of the NNC (DBM, 2020). |
| Is the government funding nutrition interventions for children? | Yes. Public health management includes integrated public health program strategies for children’s health development programs (DOH, 2018). |
| Is the government funding obesity prevention research? | Information limited. There is Php 6.78 Billion to control the burden of Non-Communicable Diseases, but the allocation for research on obesity prevention is not specified (DOH, 2019). |
| Is there a standard procedure to assess the impact of food-related policies on health and obesity? | Yes. The Government Program Participation Survey of DOST-FNRI provides information on the extent of participation of households and individuals to selected health and nutrition-related programs (DOST-FNRI, 2016). |
| Are there any monitoring reports of HIAs? | No monitoring reports available yet. |
| Are there any monitoring reports of food company activities on HFSS food promotion? | No data available. |
| ***Health System: During pregnancy*** | |
| Are adolescent and maternal nutrition programmes provided? | Yes. It is included in the PPAN 2017-2022 (NNC, 2017). Dietary supplementation guidelines for pregnant and lactating women are being finalized by the NNC. |
| Do women receive antenatal monitoring to prevent excessive weight gain and maternal diabetes? | Yes. The Department of Health (DOH) issued Administrative Order 2008-0029 titled: “Implementing Health Reforms for the Rapid Reduction of Maternal and Neonatal Mortality”. One of the essential antenatal care services provided is the monitoring of height and weight during pregnancy (DOH, 2011). The 2016 DOH administrative order (2016-0035), “Guidelines on the Provision of Quality Antenatal Care in All Birthing Centers and Health Facilities Providing Maternity Care Services”, aims to “improve the quality of antenatal care (ANC) through the provision of technical guidance in the shift in ANC concept from the high-risk approach to the four-visit model of focused ANC” (DOH, 2016). |
| Is there provision for prenatal counselling and care (including diet, physical activity and smoking)? Is this targeted at fathers as well as mothers? | Yes. Included in the IEC/Counselling on Healthy Lifestyle is counselling on smoking cessation, healthy diet and nutrition, and physical activity (DOH, 2011). |
| Are protein and/or energy supplements (or cash or food vouchers) provided to pregnant women? Is their use monitored to prevent unintentional excessive weight gain? | Government community health facilities provide pregnant women with micronutrient supplements such as iron and folic acid, and Zinc. The MNCHN Strategy Manual of Operations does not mention protein/energy supplements given to pregnant women (DOH, 2011). |
| ***Health and Social Support systems: During infancy and young childhood*** | |
| Are there programmes and practices to promote breastfeeding | Yes. RA 10028 - Expanded Breastfeeding Promotion Act of 2009. The purpose of this act is to provide incentives to all government and private health institutions with rooming-in and breastfeeding practices. The Philippine IYCF 2030 Strategic Planning was also finalized by UNICEF and the DOH in 2019. Its mission is to: 1. nurture and nourish the mother and child; 2. reduce stunting and all other forms of malnutrition, and 3. create an enabling environment for optimal infant and young child feeding. |
| Are hospitals following the recommendations of the Baby-Friendly Hospitals Initiative? | Yes. The Philippines launched the Mother-Baby Friendly Hospital Initiative (MBFHI) in 1992. Currently, according to the DOH, the certification and/or accreditation as Mother-Baby Friendly is now a requirement for hospital licensing. |
| Do women have a right to maternity leave? | Yes. RA No. 11210 or “105-day Expanded Maternity Leave Law” where women have a right to a 105-day maternity leave, with an option to extend it to 30 days without pay, and another 15 days for solo mothers. |
| Is the International Code on the Marketing of Breast-milk Substitutes implemented by national legislation? | Yes. Through Executive Order 51, or the “Philippine Milk Code", the Philippines recognizes the international code on the marketing of breastmilk substitutes. |
| Is there screening to monitor infants and young children for overweight or other malnutrition? | Yes. Operation Timbang Plus is an annual mass weighing of all pre-schoolers 0-71 months old in a community to identify and locate the malnourished children (NNC, 2012). |
| Are there policies or programmes to support families' access to healthy foods? | Yes, but limited. The National Food Authority (NFA) administers a subsidized rice program for assisting households in times of difficulty (Presidential Decree No. 4 Series of 1972). Members of Pantawid Pamilyang Pilipino Program (4Ps) receive an additional 600 pesos per month per household as a rice assistance/subsidy (DSWD, 2017).Support is provided to the sugarcane, pig meat, and poultry industries through high import tariffs (OECD, 2020). |
| Are there well-publicized referral routes for infants and young children at higher risk of overweight or obesity? | None. |
| Do primary health-care workers receive training in providing nutrition counselling? | Yes. There are training Regulations (TR) for barangay health workers (BHWs). The BHWs are volunteer community workers who identify, monitor and refer the malnourished to appropriate service providers (TESDA, 2019).The Promote Good Nutrition component of the Accelerated Hunger-Mitigation Program includes community-based training on infant and young child feeding and nutrition (NNC, 2013). The Nutrition Center of the Philippines (NCP) facilitates the recruitment and training of community-based peer counsellors, enrolled in the DOH's BFTSEk program (Breastfeeding Tama Sapat Eksklusibo) (NCP, n.d.). |
| Do new parents have access to parenting courses? | Yes. Through the Department of Social Welfare and Development (DSWD) Administrative Order No. 39 s. 2003 - Guidelines on the Implementations of Family Life Development Program. |
| ***Education System*** | |
| Are there nutritional standards for food provided to young children in child-care settings? Are there physical activity standards for child-care settings? Is there national guidance for screen-time and sleep-in child-care settings? | Yes. The Early Childhood Care and Development Law (Republic Act 8980) aims to expand nutrition access and coverage to all five-year old children (UNESCO, 2006). The DSWD has guidelines for supplemental feeding in day-care centers (DSWD A.O. No. 04, Series of 2016). |
| Are there nutrition standards for meals provided in schools? | Yes. The “Policy and Guidelines on Healthy Food and Beverage Choices in Schools and DepEd Offices” (Department Order No. 13, Series of 2017) and the “Operational Guidelines on the Implementation of School-Based Feeding Program for School Years 2017-2022” (Department Order 39, Series of 2017). In response to COVID-19, the DepEd issued the Department Order No. 023, Series of 2020 (“Operational guidelines on the implementation of the school-based feeding program for the school year 2020-2021”) which stipulates that nutritious food products for 60 feeding days and fresh or sterilized milk for 50 feeding days will be delivered to the respective homes of all beneficiaries. |
| Are these standards linked to the national FBDGs? Do they apply to all schools, private and state-run? Are they monitored and reported? | Yes. They are linked to the dietary guidelines set by the DOST-FNRI. |
| Are there nutrition standards for other foods (snacks and beverages) sold in schools, e.g. in vending machines? | Yes. DepEd Administrative Order no. 13 Series of 2017 - food products with nutrition labels are grouped into green, amber, and red categories, which dictates their availability. Canteens are also encouraged to follow the Pinggang Pinoy guidelines for meal plans and menus. |
| Are there standards for children’s physical activity? | Yes. The Philippine National Guidelines on Physical Activity included in the 2010 publication of the Department of Health’s “HEALTHBeat”, prescribed at least 60 minutes of daily exercise for children aged 5-12 years old and young adults aged 13 to 20 years old. |
| Are there standards for the amount of sedentary time, or screen time, for children at school? Does this cover all schools, state and private? | No data available. |
| Are there policies to ensure access to safe drinking water in schools and sport facilities? | Yes. There is a program called “WASH in Schools: Three stars approach” where the procedures in ensuring quality of water served in schools is stated (Department of Education, 2018). |
| Are there policies to encourage active travel (walking and cycling) to and from school? | No data available. |
| Are there policies to give access to school and municipal sport and play facilities outside of school hours? | There is no specific policy to give access to school and municipal sport and play facilities outside of school hours. Access is determined at a school-level. The Educational Facilities Manual stipulates that provisions shall be considered for the use of school and municipal sport and play facilities by the community during off-school hours (DepEd, 2010). |
| Are there school-based fitness monitoring programmes? | Yes. There is a Physical Fitness Test which is a set of measures designed to determine a learner's level of physical fitness. Results are recorded, summarized, and submitted to the Bureau of Curriculum Development-Special Curricular Programs Division (BCDSCPD) for evaluation (DepEd Order No. 034, Series of 2019 or the “Revised Physical Fitness Test Manual”) |

Supplementary File 3E: Environments and policies that determine the risk factors for overweight and obesity in Vietnam

| **Key questions** | **Information** |
| --- | --- |
| ***National policy*** | |
| Does the national government have a written policy addressing obesity? Does this specifically include childhood obesity? Do any of the policy statements include targets (infants, under 5s, and children)? | Yes. National target with child overweight less than 5% in rural areas and less than 10% among urban population |
| Do any of the policy statements discuss double burden or double-duty actions to tackle obesity as part of a wider malnutrition/hunger strategy? | Yes. By setting the target for both malnutrition and overweight, as well as target in improving healthy eating and physical activities |
| ***Food Based Dietary Guidelines and Nutrient Profiling Schemes*** | |
| Are there government-endorsed national Food Based Dietary Guidelines (FBDGs) and do these include specific recommendations for children e.g. infants, young children, or adolescents? | Yes, Pyramid of Health and 10 tips on reasonable nutrition to 2020 |
| Is there a government-endorsed nutrient profiling scheme (NPS), e.g., used to restrict marketing foods to children or to classify foods for front-of-pack labelling signals? | No. |
| Are there specific subsidies, taxes, or levies on foods or beverages linked explicitly to the government endorsed FBDGs or NPS? | No. There is the proposed law of applying 10% tax on sugar and carbonated soft drink, but it has not been approved |
| Are there any examples of government procurement policies linked specifically to FBDGs or NPS? | No. General quality standards, not nutrition-based |
| Are there any local or national controls on commercial catering services (including fast food chains) linked specifically to FBDGs or NPS? e.g. restrictions on allowing fast food stores near schools? | Yes. But only on school campus |
| ***Food Labelling and Marketing*** | |
| Is there a government-endorsed front-of-pack nutrition labelling scheme which signals foods high in fats or sugars? Menus: are there policies to require food service operators to show nutritional information on their menus? | Side of pack ingredients and nutrient lists. No front of pack restrictions |
| Are there policies to control what types of food and beverages are being promoted: on TV? in the street? on digital media? | General rules on truthful advertising, not specific for nutrition |
| Are chain restaurants/fast food stores offering healthy versions of popular products (especially those for children)? | Yes. But it is voluntary |
| ***Food reformulation, taxation, subsidy, and research*** | |
| Food composition/reformulation - Are there government-set targets for reducing salt, fat, or sugar for health purposes? | No government targets |
| Is there a policy to subsidise or encourage (e.g., through market support or tax relief) home-produced foods which meet nutrition criteria, e.g., government endorsed FBDGs or NPS? | No strong linking between agriculture production and FBDGs, but support for more fresh produce |
| Is there a policy to subsidise or support food chain processes (transport, warehousing, chilling, freezing) specifically linked to government endorsed FBDGs or NPS criteria? | No linking |
| Is there a policy for restricting imports of foods that fail to meet specific nutritional criteria, e.g., using FBDGs or NPS classification? | No linking |
| Is there a policy to support research or outreach training and development that specifically links to government endorsed FBDGs or NPS criteria? | No strong linking between agriculture development and FBDGs, but support for more fresh produce |
| ***Environmental and cultural factors*** | |
| Are local water supplies believed to be safe to drink? Is water widely available? | Yes. Except for some rural areas |
| Are there specific cultural norms that reduce the opportunities to take physical activity? Or increase the need for sedentary behaviour? | Boys prefer more intensive sport (e.g., football, badminton…) than girls, but there is no restriction |
| Does the prevailing climate affect dietary behaviour or physical activity? Does the natural terrain affect physical activity? | Yes, in some extreme cases (e.g., extremely hot or cold in the North) but it is not significant |
| Are there policies at national or city level to provide safe cycling and walking routes? | No cycle lanes |
| Are there policies at national or city level to reduce car use? This includes policies to improve air pollution as well as encourage outdoor activity. | No car restrictions |
| Is there evidence of a code of conduct for media companies, or for journalists, on reporting on obesity and avoiding stigma and victim blaming? | There is no evidence of obesity stigma in males, but some in females |
| Are there cultural preferences for particular foods or cooking practices? | There is no linking with FBDGs |
| Are there cultural reasons why fatness might be encouraged? Are these changing? Are there gender differences? | Assumption that fatter baby is healthiest |
| Are there specific rules about feasting or fasting which may encourage weight gain? | No specific rule. |

Supplementary File 3F: Environments and policies that determine the risk factors for childhood overweight and obesity in Vietnam

| **Key questions** | **Information** |
| --- | --- |
| ***Level of National Support*** | |
| Is there visible support from the president/prime minister/cabinet office for action on childhood obesity? | No specific minister. Department of Preventive Medicine – Ministry of Health (MoH) is responsible for managing NCDs, but the role in childhood overweight is not clear. Recognises links to undernutrition. |
| Is there an obesity policy coordination platform for national and local government collaboration? or for government and NGO collaboration? | No obesity body. National institute of nutrition, Department of Children - Mother Health, Ministry of Education and Training |
| Are there city-led or local authority-led strategies and policies to tackle child obesity? | School food and PA (sport competitions) set at local authority or national level |
| Does the government (or other independent organisation) conduct surveillance to monitor child obesity levels, and report the results? or monitor diet and nutrition intakes? | No specific monitoring program |
| Does the government have obesity (and undernutrition) targets? Are they time-limited? | WHO/UNICEF targets |
| Is there monitoring of the media narrative on child weight, etc? | No media monitoring. There are some tips published for healthy child’s 1^st^ year, tips for healthy pregnant, 10 tips on reasonable nutrition … |
| Is there good public access to government information and the evidence used for obesity-related policy-making? | Limited documentation and hard to access by lay people |
| Is the government funding nutrition education promotion programs? | Limited, mainly for malnutrition. Funding for overweight/obesity is a small part of the package |
| Is the government funding nutrition interventions for children? | Limited, mainly for malnutrition |
| Is the government funding obesity prevention research? | No |
| Is there a standard procedure to assess the impact of food-related policies on health and obesity? | No |
| Are there any monitoring reports of HIAs? | No |
| Are there any monitoring reports of food company activities on HFSS food promotion? | No |
| ***Health System: During pregnancy*** | |
| Are adolescent and maternal nutrition programmes provided? | Healthy eating in pregnancy taught antenatal classes, but limited in the urban areas |
| Do women receive antenatal monitoring to prevent excessive weight gain and maternal diabetes? | Yes, when attending antenatal clinics |
| Is there provision for prenatal counselling and care (including diet, physical activity and smoking)? Is this targeted at fathers as well as mothers? | Yes, when attending antenatal clinics |
| Are protein and/or energy supplements (or cash or food vouchers) provided to pregnant women? Is their use monitored to prevent unintentional excessive weight gain? | Yes. IFA |
| ***Health and Social Support systems: During infancy and young childhood*** | |
| Are there programmes and practices to promote breastfeeding | Department of Children - Mother Health and NGOs (Alive and Thrive) |
| Are hospitals following the recommendations of the Baby-Friendly Hospitals Initiative? | Yes, has been implemented in most areas |
| Do women have a right to maternity leave? | Yes, 6 months |
| Is the International Code on the Marketing of Breast-milk Substitutes implemented by national legislation? | Partially, advertisement and package label must include the statement that breast milk is the best food for infants |
| Is there screening to monitor infants and young children for overweight or other malnutrition? | Yes, when attend paediatric clinics |
| Are there policies or programmes to support families' access to healthy foods? | No |
| Are there well-publicized referral routes for infants and young children at higher risk of overweight or obesity? | No specific pathways |
| Do primary health-care workers receive training in providing nutrition counselling? | Yes, but not specialize on overweight/obesity mainly for malnutrition |
| Do new parents have access to parenting courses? | No, unless private |
| ***Education System*** | |
| Are there nutritional standards for food provided to young children in child-care settings? Are there physical activity standards for child-care settings? Is there national guidance for screen-time and sleep-in child-care settings? | Pre-school meal standard for  No specific standard for PA or sleep time |
| Are there nutrition standards for meals provided in schools? | Up to local education authority.  No specific nutrition standards for meals provided in schools, PA or sedentary time |
| Are there standards for children’s physical activity? | Only in school with minimum hours |
| Are there standards for sedentary time, or screen time, for children at school? Does this cover all schools, state and private? | No |
| Is nutrition education included on the school curriculum? | Limited, available in some schools as extracurricular |
| Are there policies to ensure access to safe drinking water in schools and sport facilities? | Yes, in schools |
| Are there policies to encourage active travel (walking and cycling) to and from school? | No, but encourage active travel |
| Are there policies to give access to school and municipal sport and play facilities outside of school hours? | Up to each school and local authority |
| Are there nutrition standards for other foods (snacks and beverages) sold in schools, e.g., in vending machines? | No |
| Are there school-based fitness monitoring programmes? | No |

**Supplementary File 4A. Overweight and obesity amongst children in Mongolia aged <5 years, by income quintile and mother’s education**

| **Indicator** | **Under 5 y/o**  **(overweight-for-height)** | |
| --- | --- | --- |
|  | **Value** | **Rating scale^1^** |
| ***Income quintile*** | | |
| Quintile 1 (lowest income) ^2^ | 10.8% | High |
| Quintile 2 ^2^ | 13.6% | High |
| Quintile 3 ^2^ | 7.5% | Medium |
| Quintile 4 ^2^ | 11.8% | High |
| Quintile 5 (highest income) ^2^ | 9.3% | Medium |
| ***Mother’s education*** | | |
| None | 10.2% | High |
| Primary | 11.7% | High |
| Some secondary) | 10.7% | High |
| Completed secondary | 10.0% | High |
| Vocational training | 8.4% | Medium |
| College/university | 11.1% | High |

*^1^Lobstein & Jewell. (2021). What is a "high" prevalence of obesity? Two rapid reviews and a proposed set of thresholds for classifying prevalence levels, Obesity Reviews. DOI: 10.1111/obr.13363*

*^2^Mongolia Social indicator sample survey – 2018 detailed results*

**Supplementary File 4B. Overweight and obesity amongst children in the Philippines aged <5 years, 5-10 years, and 10-19 years in 2019, by income quintile**

| **Income quintile** | **Under 5 y/o**  **(overweight-for-height)** | | **5-10 y/o**  **(overweight/obesity)** | | **10-19 y/o**  **(overweight/obesity)** | |
| --- | --- | --- | --- | --- | --- | --- |
|  | **Value** | **Rating scale^1^** | **Value** | **Rating scale^1^** | **Value** | **Rating scale^1^** |
| Quintile 1 (lowest income) ^2^ | 1.0% | Very Low | 1.9% | Very Low | 3.8% | Low |
| Quintile 2 ^2^ | 1.5% | Very Low | 4.7% | Low | 5.2% | Medium |
| Quintile 3 ^2^ | 2.3% | Very Low | 7.9% | Medium | 8.6% | Medium |
| Quintile 4 ^2^ | 5.0% | Medium | 15.2% | Very High | 14.0% | High |
| Quintile 5 (highest income) ^2^ | 8.0% | Medium | 26.5% | Very High | 23.0% | Very High |

*^1^Lobstein & Jewell. (2021). What is a "high" prevalence of obesity? Two rapid reviews and a proposed set of thresholds for classifying prevalence levels, Obesity Reviews. DOI: 10.1111/obr.13363*

*^2^DOST-FNRI. (2020) Expanded National Nutrition Survey: 2019 Results Retrieved from* [*http://enutrition.fnri.dost.gov.ph/site/presentation.php?year=2019*](http://enutrition.fnri.dost.gov.ph/site/presentation.php?year=2019)

**Supplementary File 5: Identified priority policies for addressing childhood overweight and obesity in Mongolia and the Philippines**

| **Mongolia** | **The Philippines** |
| --- | --- |
| ***Overarching actions*** | |
| Establish a cross-government nutrition council on prevention of overweight and obesity, excluding commercial interests. | Develop a strategy to address overweight and obesity through multiple sectors and systems. |
| Drive social and behaviour change to create awareness and improve nutrition and physical activity practices. | Drive social and behaviour change to create awareness and improve nutrition and physical activity practices. |
| Advocate and build capacity of policymakers on the causes of childhood overweight and relevant actions for its prevention. | Improve data collection and reporting on overweight and obesity through surveys and routine data. |
| ***Food system*** | |
| Introduce legislation to prohibit unhealthy food advertising to children | Introduce legislation to prohibit unhealthy food advertising to children |
| Introduce and impose statutory sanctions to control the promotion of highly processed, unhealthy foods. | Monitor and enforce the existing Philippine Milk Code. |
| Implement a mandatory front-of-pack label on high fat, salt and sugar foods. | Monitor and enforce the existing regulation on the marketing of unhealthy food and beverages in schools |
| Implement an industry levy or enhanced sales tax on sugary beverages and snack foods. | Implement a mandatory front-of-pack label on high fat, salt and sugar foods. |
| Reformulate processed foods to reduce fat, sugar and salt, and portion sizes. | Provide subsidies/ incentives and infrastructure to food producers to increase availability and access to healthier foods. |
| Support nutrition sensitive procurement policies. | Improve the quality of complementary foods ensuring adherence to set national standards and guidelines. |
| ***Health system*** | |
| Strengthen the implementation of the Code of Marketing of BMS and the ‘10 steps for successful breastfeeding’ in health facilities. | Strengthen systems for screening, referral, counselling and care to prevent and manage overweight among women and children. |
| Strengthen nutrition counselling and weight control in antenatal care. | Include overweight and obesity prevention and management in universal health care coverage. |
| Strengthen healthy eating and nutrition counselling for parents on prevention and control of childhood overweight and obesity | Strengthen the capacity of health care providers to deliver overweight and obesity interventions |
| ***Education system*** | |
| Develop and implement food and nutrition standards for preschool and school settings. | Implement, enforce, and monitor existing food and nutrition standards for preschool and school settings. |
| Develop and implement physical activity standards for preschool and school settings. | Enhance the school curricula to include nutrition and physical activities. |
| Monitor child growth in all education settings. | Strengthen the capacity of teachers to deliver interventions that promote healthy school food environments. |
| Support nutrition literacy and physical education in preschool and school settings. |  |
| ***Environment, and water, sanitation and hygiene systems*** | |
| Promote convenient, safe, and connected walking and cycling infrastructure and reduce car use in urban plans. | Promote convenient, safe, and connected walking and cycling infrastructure and reduce car use in urban plans. |
| Improve access to free, safe and potable drinking water in schools and local communities. | Improve access to free, safe and potable drinking water in schools and local communities. |
| ***Social protection system*** | |
| Enhance social protection policies to deliver healthy and nutritious food packs. | Enhance social protection policies to deliver healthy and nutritious food packs. |
| Improve facilities for breastfeeding mothers in public and at workplaces. | Use social protection programs to improve access to healthy diets and drive social behaviour change. |

**References**

1. United Nations Children’s Fund, World Health Organization, The World Bank. UNICEFWHO-World Bank Joint Child Malnutrition Estimates. UNICEF, New York; WHO, Geneva; The World Bank, Washington; 2012.

2. Abarca-Gómez L, Abdeen ZA, Hamid ZA, Abu-Rmeileh NM, Acosta-Cazares B, Acuin C, et al. Worldwide trends in body-mass index, underweight, overweight, and obesity from 1975 to 2016: a pooled analysis of 2416 population-based measurement studies in 128.9 million children, adolescents, and adults. The Lancet. 2017;390(10113):2627-42.

3. World Health Organization. Global School-based Student Health Survey Geneva2022 [20 January 2022]. Available from: <https://www.who.int/teams/noncommunicable-diseases/surveillance/systems-tools/global-school-based-student-health-survey>.

4. United Nations Children’s Fund, World Health Organization. Indicators for assessing infant and young child feeding practices. Geneva; 2021.

5. United Nations Children’s Fund, World Health Organization. UNICEF-WHO Low birthweight estimates: Levels and trends 2000–2015. Geneva; 2019.

6. World Health Organization. The Global Health Observatory Geneva: World Health Organization; 2022 [20 January 2022]. Available from: <https://www.who.int/data/gho>.

7. World Health Organization, United Nations Children’s Fund. WHO/UNICEF Joint Monitoring Programme for Water Supply, Sanitation and Hygiene 2021 [Available from: <https://washdata.org/>.

8. WHO Multicentre Growth Reference Study Group. WHO Child Growth Standards based on length/height, weight and age. Acta paediatrica (Oslo, Norway : 1992) Supplement. 2006;450:76-85.
